# A Plausible Role for Collectins in Skin Immune Homeostasis

**DOI:** 10.3389/fimmu.2021.594858

**Published:** 2021-03-15

**Authors:** Tian Wang, Ke Li, Shengxiang Xiao, Yumin Xia

**Affiliations:** ^1^ Department of Dermatology, The Second Affiliated Hospital of Xi’an Jiaotong University, Xi’an, China; ^2^ Core Research Laboratory, The Second Affiliated Hospital of Xi’an Jiaotong University, Xi’an, China

**Keywords:** collectins, immunity, immune homeostasis, skin, immunotherapy

## Abstract

The skin is a complex organ that faces the external environment and participates in the innate immune system. Skin immune homeostasis is necessary to defend against external microorganisms and to recover from stress to the skin. This homeostasis depends on interactions among a variety of cells, cytokines, and the complement system. Collectins belong to the lectin pathway of the complement system, and have various roles in innate immune responses. Mannose-binding lectin (MBL), collectin kidney 1, and liver (CL-K1, CL-L1) activate the lectin pathway, while all have multiple functions, including recognition of pathogens, opsonization of phagocytosis, and modulation of cytokine-mediated inflammatory responses. Certain collectins are localized in the skin, and their expressions change during skin diseases. In this review, we summarize important advances in our understanding of how MBL, surfactant proteins A and D, CL-L1, and CL-K1 function in skin immune homeostasis. Based on the potential roles of collectins in skin diseases, we suggest therapeutic strategies for skin diseases through the targeting of collectins and relevant regulators.

## Introduction

The skin is one of the largest organs in mammals, and it plays a significant role in innate immunity. It serves not only as a physical barrier to protect the host from mechanical injury, ultraviolet irradiation, and environmental toxins, but also as an immunological barrier against a variety of pathogens. Skin immune homeostasis refers to the process of defending against external microorganisms, while at the same time clearing apoptotic cells, and promoting the recovery of tissues damaged by wounds, inflammation, and diseases (1). Innate immune mechanisms orchestra skin immune homeostasis, which rely on sophisticated networks of different cell types, the participation of cytokines, and tight regulation of the complement system activation.

Collectins belong to the C-type lectin superfamily, defined by structural inclusion of a collagen-like region and a C-type lectin domain, also referred to as the carbohydrate recognition domain (CRD) (2). Collectins play an important role in innate immunity. They were found to recognize pathogen-associated molecular patterns, mediate pathogen clearance, modulate immune cells in tissues as well as initiate lectin pathway (3). Collectins have an important role in eliminating microorganisms from skin (4). MBL, a member of collectins family, participates in skin apoptotic cells clearance (5). Abnormal levels of collectins are seen during skin diseases (6, 7). Together, these findings suggest that collectins have an important role in skin immune homeostasis.

## Skin Immune Homeostasis With Focus on Innate Immune Mechanisms

### Nonimmune Cells

**Figure 1:**
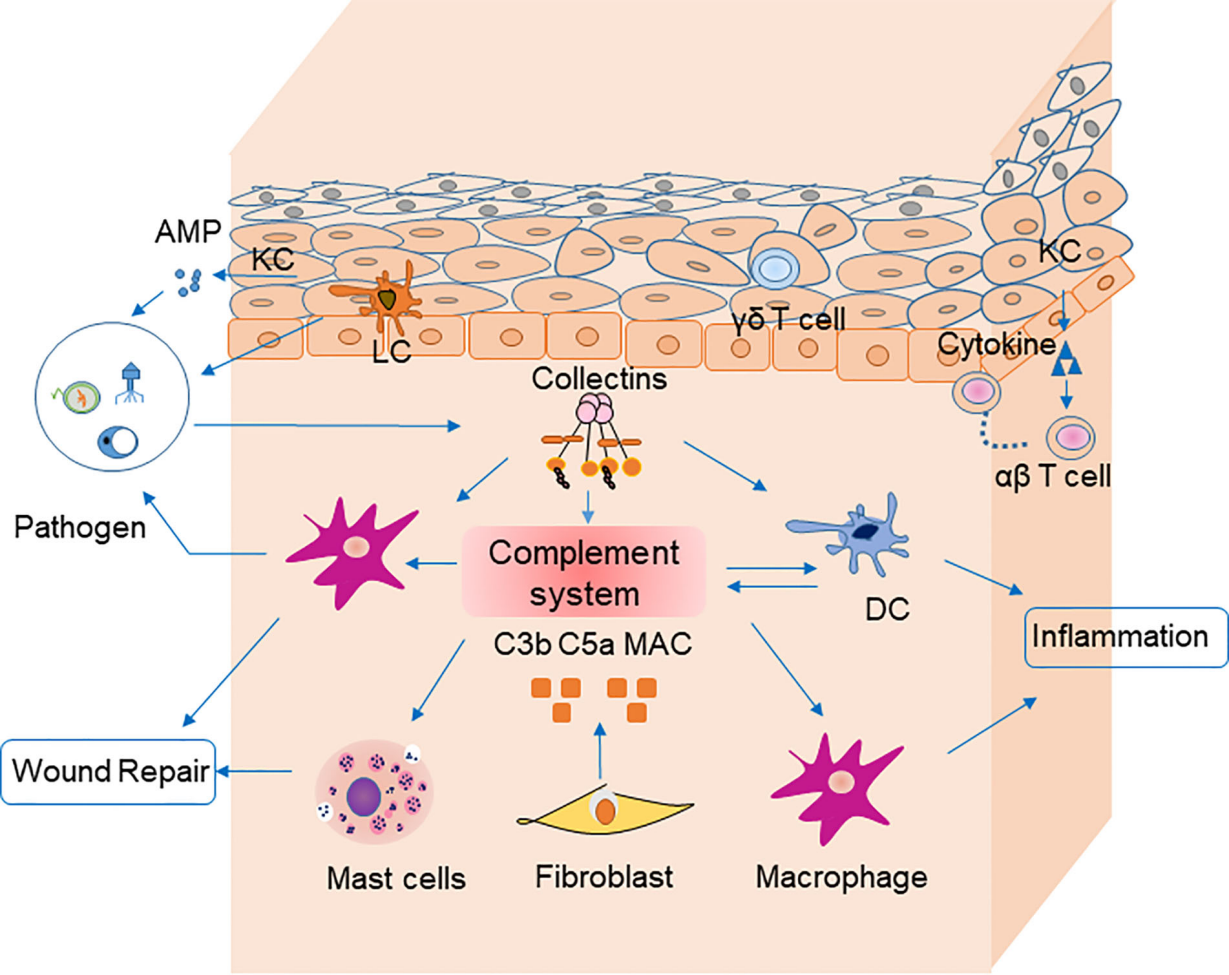
Cells, cytokines, and the complement system are involved in skin homeostasis. Keratinocytes recognize pathogen-associated molecular patterns *via* pattern recognition receptors on their surfaces, and they secrete antimicrobial peptides. Fibroblasts secrete C3b, C5a, and MAC, which are involved in pathogen clearance by complement. Langerhans cells are antigen-presenting cells for microbial antigens in the epidermis. Dendritic cells secret most components of complement system and mediate together with macrophages pahgocytosis of pathogens *via* complement receptors (CR1, CR3 and CR4). They could mediate phagocytosis in the clearance of pathogens by receptors (e.g., C3R, C4R) on their surface in turn. Macrophages participate in inflammation and wound repair. Receptors for C3a on mast cells can be activated by their ligands to degranulate and secret cytokines including MCP-1 and RANTES, which participate in wound repair. Pathogens stimulate collectins to activate complement system. Collectins may also modulate inflammation by macrophages and DCs directly. Keratinocytes recruit αβ T cells, and γδ T cells to epidermis. AMP, antimicrobial peptide; PRR, pattern recognition receptor; APC, antigen-presenting cell; DC, dendritic cells.

Several nonimmune cells participate in skin immune homeostasis. Keratinocytes secrete antimicrobial peptides and various cytokines, such as interleukin (IL)-6, IL-1β, and TNF-α, all of which activate inflammation and recruit neutrophils and macrophages (8). Keratinocytes recognize pathogen-associated molecular patterns *via* pattern recognition receptors, such as toll-like receptors, on their cell surfaces (9). Melanocytes also produce cytokines involved in leukocyte recruitment (10). Fibroblasts express toll-like receptors and suppress T cell proliferation (11). These nonimmune cells also influence inflammation by secretion of complement components (12) (**Figure 1**).

### Resident Myeloid Cells

In the epidermis, Langerhans cells that process and present microbial antigens are most prevalent. Langerhans cells migrate to skin-draining lymph nodes, where they stimulate T lymphocytes (13). Antigen-presenting cells, such as macrophages, dendritic cells, and mast cells, reside in the dermis, which is the middle layer of the skin. Macrophages have a variety of homeostatic roles in wound repair, follicle regeneration, salt balance, and even tumor immunity (14). Dendritic cells are relevant to immunosurveillance during inflammatory responses (15). Mast cells function in inflammation and allergic reactions by secreting histamines. The aforementioned cells are also involved in skin immunity *via* the complement system. Dendritic cells produce varieties of complement components including C1q, C3, Factor I and Factor B (16). Receptors on dendritic cells (e.g., C3R, C4R) bind inactivated fragments of C3 (iC3b) and mediate phagocytosis in the clearance of pathogens (17). G-coupled receptors for C5a and C3a on mast cells can be activated by their ligands to degranulate and secret cytokines including MCP-1 and RANTES, which participate in wound repair (**Figure 1**) (18, 19).

### Infiltrating Immune Cells

Many cells infiltrate the skin during immune responses. The total quantity of cutaneous T cells is estimated to be nearly twice that in the blood (20). Resident memory T cells, belonging to αβ T lymphocytes, are located in both the epidermis and dermis (21). Most accumulate in the skin as a result of inflammation, and they persistently recruit other memory T cells (22). They interact with major histocompatibility complexes that present peptides. On the other hand, γδ T lymphocytes lack the ability to recognize major histocompatibility complex-restricted peptides but are also present in the skin (23). γδ T produces antimicrobial peptides against microorganisms, secrete growth factors during wound repair, and inhibit tumor growth in the skin (24, 25). B lymphocytes and neutrophils are rare in normal skin but common in cutaneous diseases (26). The innate-like subset of B lymphocytes take part in autoimmunity, inflammation and infection by producing IL-10 (27). Neutrophils infiltrate infected skin and produce substances to recruit more immune cells. Lastly, eosinophils express eosinophil peroxidase and other substances that are important regulators in allergic skin diseases.

### Complement System

The complement system consists of plasma proteins, cell surface complement receptors, and regulatory proteins found both on the surface of cells and solution. Most complement components are synthesized by hepatocytes, but some are also synthesized macrophages, dendritic cells, fibroblasts and keratinocytes in the skin (12). The complement pathway is also regulated by cytokines. For example, C3 synthesis by keratinocytes is positively related to IL-1α, interferon (IFN)-γ, and tumor necrosis factor (TNF)-α levels (28). Extensive accumulation of C3 and C4 can be reduced by factor I *via* IFN-γ (29). Therefore, complement components and regulators are closely related to cells and cytokines in the skin.

**Figure 2:**
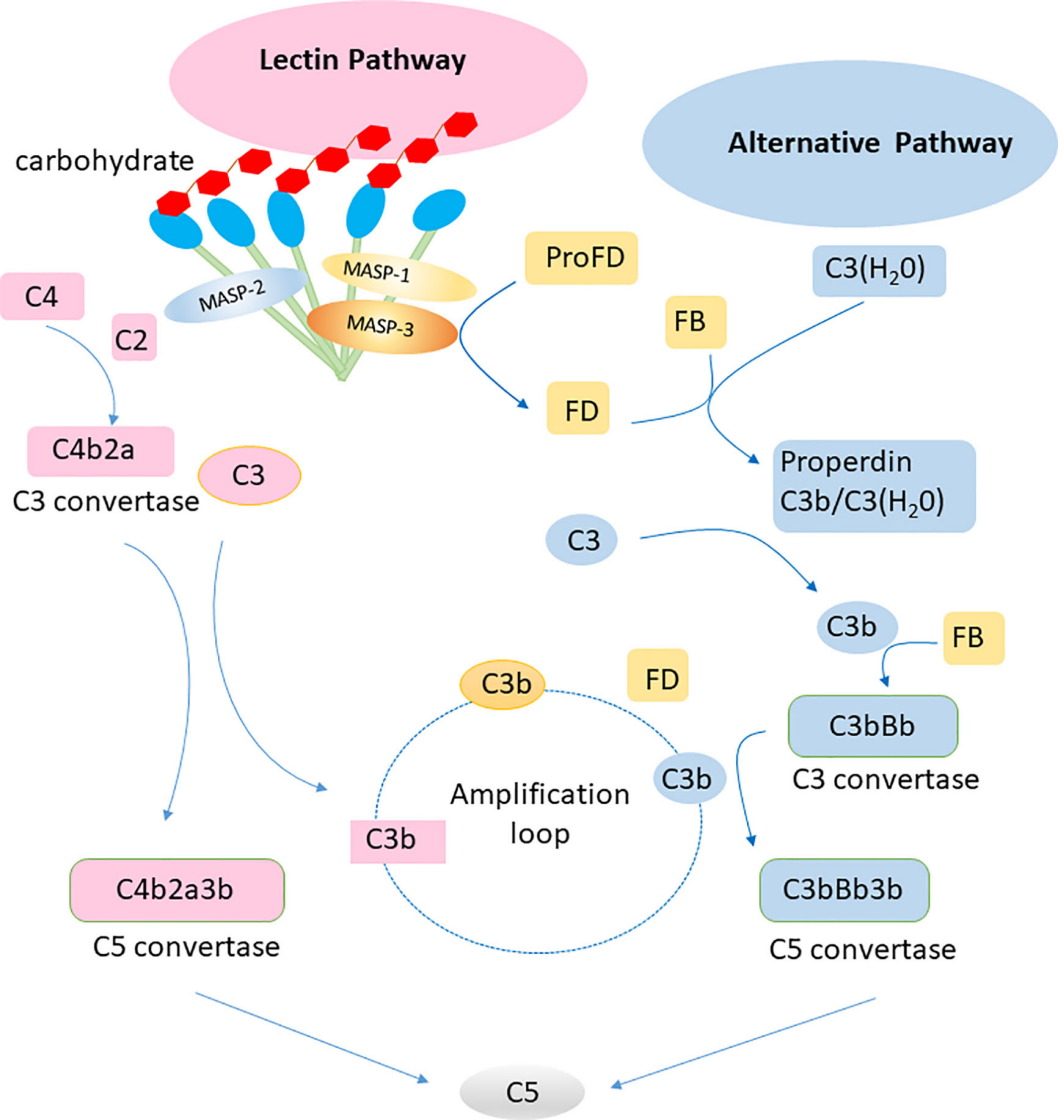
Lectin pathway and alternative pathway of the complement system. Collectins (including MBL, CL-L1, CL-K1 and the complex of the latter CL-LK complex) recognize carbohydrates on surface of pathogens and activate the lectin pathway *via* MASP-1 and MASP-2. The alternative pathway is activated by spontaneous hydrolysis of C3. Factor D is a rate limiting factor for generation of the C3 convertase in the alternative pathway, which is activated by MASP-3. The alternative pathway works as an amplification loop for the lectin pathway.

Complement can be activated *via* three pathways: classical, alternative, and lectin. The classical pathway of complement activation is initiated upon binding of the C1 complex to antibody-antigen complexes. The initiation of the alternative pathway of complement activation depends on the spontaneous hydrolysis of C3 in solution. Factor B can be activated by factor D to associate with hydrolyzed C3, leading to initiation of an amplificative loop on microbes but normally not on host cells that are protected by regulating mechanisms. The central event in the amplificative loop is the fomation of the alternative C3 convertase, C3bBb, wherein factor B, once bound to deposited C3b is activated by factor D. The lectin pathway of complement activation is initiated by the binding of collectins or ficolins to carbohydrates or acetylated residues on microorganisms or cell surfaces. Activation of the lectin pathway is mediated *via* MBL-associated serine protease 1 and 2, which cleaves C4 and C2, generating the lectin/classical C3 convertase, C4bC2a (**Figure 2**). These three pathways are different but have similar functions in inflammation, opsonization and the assembly of membrane attack complexes. Judging from the observation of polymorphisms leading to deficiencies in humans, the alternative pathway with its integrated amplificative loop appears to be the most important pathway in terms of innate immunity towards microbes. Active factor D is a dominant rate limiting factor for generation of the C3 convertase in the alternative pathway/amplificative loop (30). The accessibility of factor D is determined by MASP-3, which convert zymogen pro-factor D to “active” factor D, once the lectin pathway is activated (31). The complement system is also involved in maintaining the microbial ecosystem of the skin, e.g. through c5a-receptor 1 signaling and regulated expression of pattern recognition receptors, and proinflammatory mediators (32). Low levels of cell-bound regulators have been found in vitiligo, which illustrates the role of complement in preventing melanocyte lysis (33). Several skin diseases, including psoriasis and systemic lupus erythematosus (SLE), are associated with abnormal complement component levels or deposition. Thus, the complement system is involved in skin immune homeostasis for its functions of defending against infections, clearing immune complexes and dying cells, and linking innate and adaptive immunity *via* CD46 (alias membrane cofactor protein, MCP) on dendritic cells and T lymphocytes (34).

## Role of Collectins in Skin Immune Homeostasis

**Figure 3:**
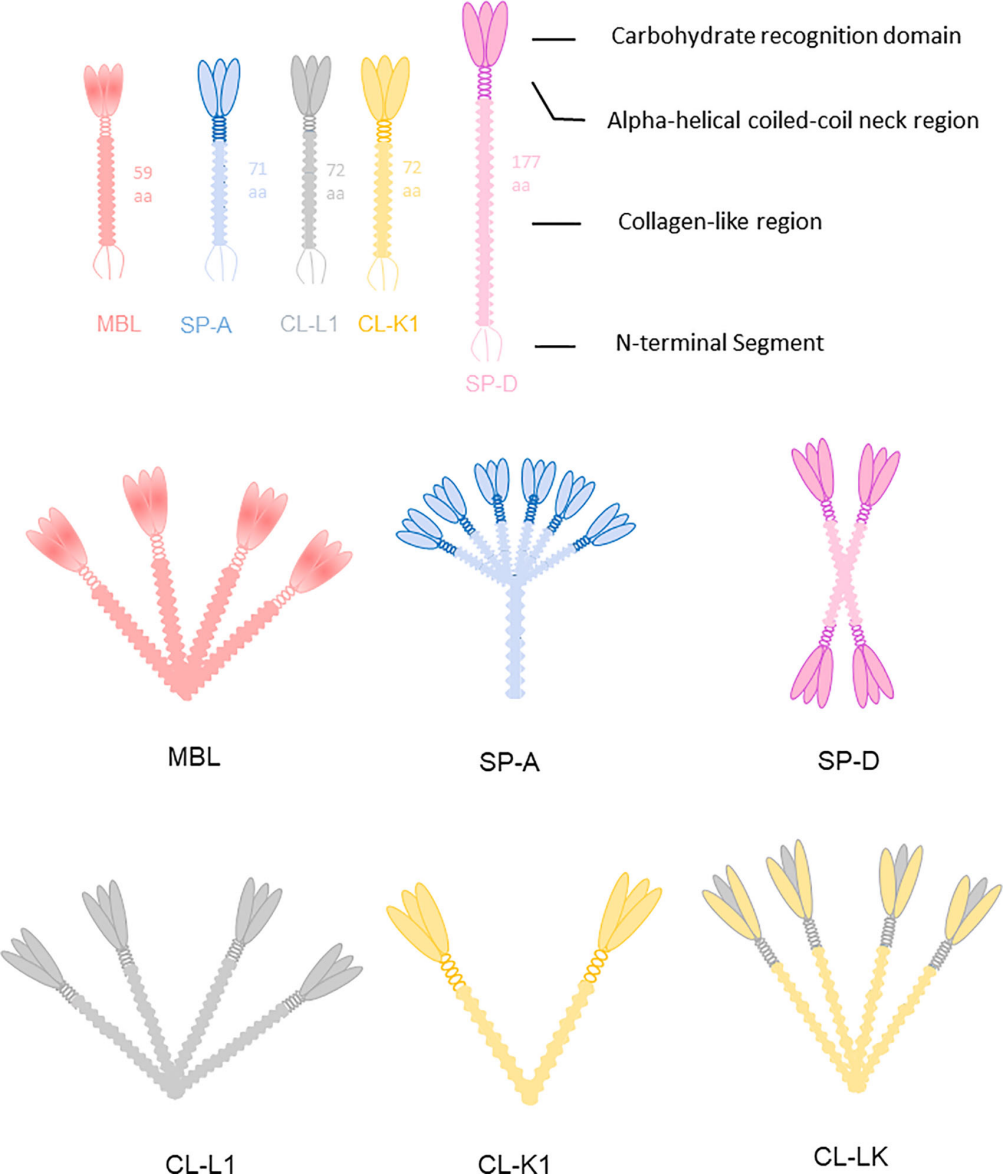
Collectins are constructed of four domains: an N-terminal cysteine-rich segment, a collagen-like region, a neck region and a C terminal carbohydrate recognition domain (CRD). MBL usually forms trimers to hexamers. CL-K1 forms dimers and trimers of trimeric subunits. SP-A and SP-D are mainly hexamers and cruciform tetramers. The subunit of CL-LK are formed by two CL-K1 and one CL-L1 approximately.

Collectins are members of the C-type lectin superfamily. To date, nine collectins have been identified: mannose-binding lectin (MBL, alias mannan-binding lectin), surfactant protein A (SP-A), surfactant protein D (SP-D), collectin liver 1 (collectin-10, CL-L1), collectin kidney 1 (collectin-11, CL-K1), collectin placenta 1 (collectin-12, CL-P1, alias scavenger receptor C-type lectin), conglutinin, and collectins of 43 kDa (CL-43) and 46 kDa (CL-46) (35). All collectins are soluble proteins except CL-P1, which is not a traditional collectin but evolved from a common ancestor of the scavenger-like receptor family, and not included further in current review. Collectins are sometimes referred to by the names of their encoding genes. For example, collectin liver 1 is encoded by *COLEC10* and is also called collectin-10. All collectins comprise a N-terminal segment with 1-3 cysteine residues, a collagen-like region of varying length, an alpha-helical coiled-coil region, and a well-conserved Ca^2+^-dependent carbohydrate recognition domain (C-type family). Collectins are assembled into subunits made of three polypeptide chains, which are organized into higher-order oligomeric structures (**Figure 3**). Most collectins include a conserved EPN motif central in the CRD, facilitating binding to D-mannose or L-fucose ligands.

As a pattern-recognition molecule, MBL functions in the lectin pathway and alternative pathway by recognizing pathogens with sugar moieties and facilitating the opsonization in both absence or presence of complements activation on the microbial surface. SP-A and SP-D were initially characterized as present in lung surfactant but later studies have demonstrated SP-A and SP-D are widespread in epithelial tissues, including the skin. Conglutinin, CL-43, and CL-46 bind to microbial glycoconjugates and conglutinin to iC3b- fragments of C3 and are only found among the Bovidae (36). Collectins are not restricted to the role of pathogen pattern recognition. In absence of microbial stimuli, they also bind to immunomodulatory receptors including SIRP-α and modulate inflammatory responses (37).

**Figure 4:**
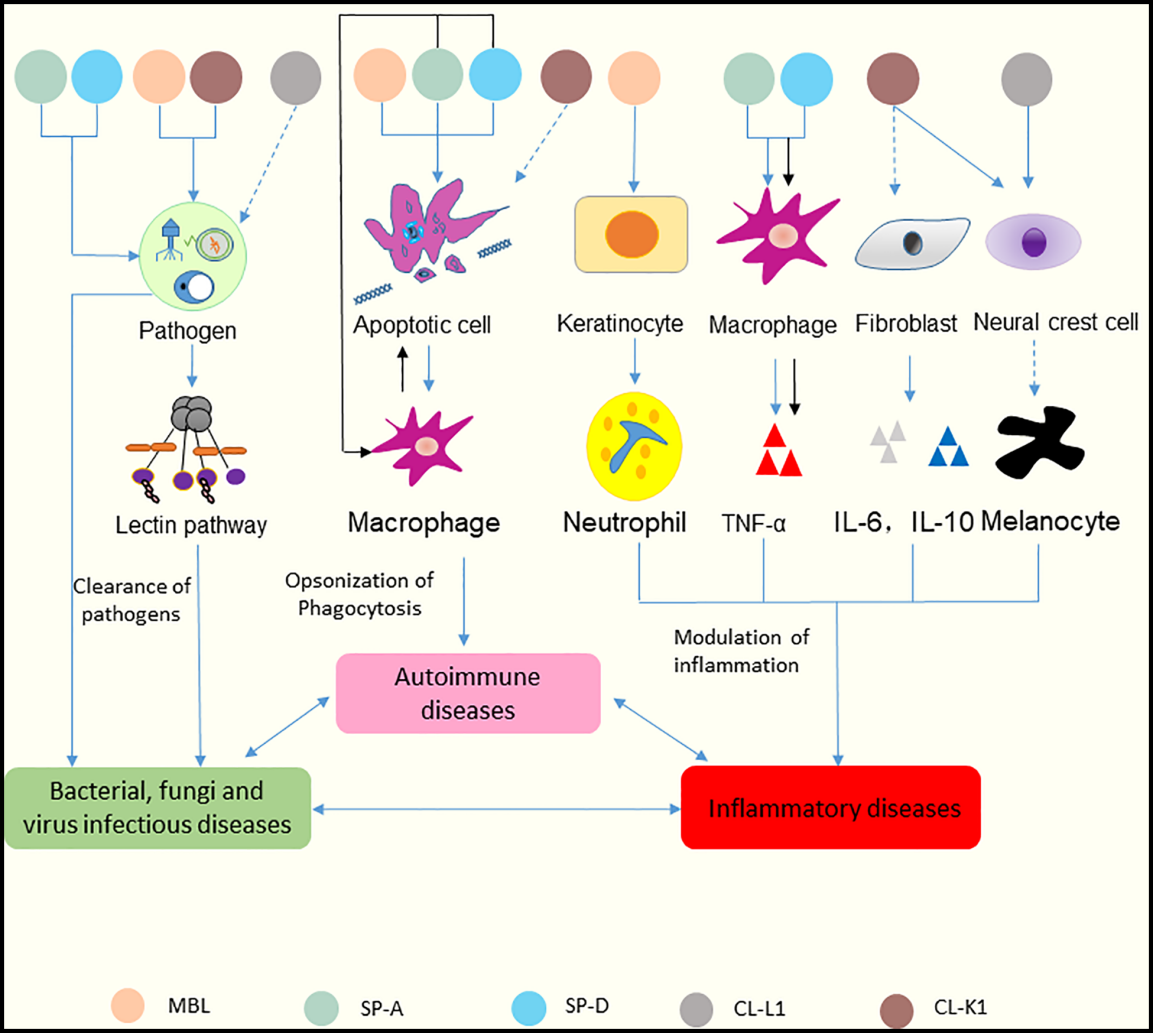
Functions of collectins in skin immune homeostasis. Collectins are similar in structure in terms of their carbohydrate recognition domains, collagen-like, and N-terminal regions. MBL and CL-K1 recognize microorganisms and activate the lectin pathway to clear pathogens. SP-A and SP-D can bind to microorganisms directly and cause aggregation. CL-L1 might initiate the lectin pathway by forming CL-LK with CL-K1. MBL, SP-A, and SP-D can recognize DNA from apoptotic cells and mediate the uptake of apoptotic cells. SP-A and SP-D also suppress apoptotic cell clearance by binding SIRP-α on macrophages (black arrows). MBL promotes keratinocytes to produce chemokine (C-X-C motif) ligand 1 to increase neutrophil infiltration. Surfactant proteins might induce the production of TNF-α *via* macrophages. SP-A and SP-D can bind to SIRP-α on macrophages and epithelia through their globular heads to suppress inflammation (black arrows). CL-K1 may modulate the production of IL-6 and IL-10 by fibroblasts. CL-K1 and CL-L1 may influence the function of melanocytes by affecting the immigration of neural crest cells. Clearance of pathogens rely on the participation of inflammatory cells and cytokines, and inflammatory responses lead to immune cells undergoing apoptosis. In reverse, accumulation of apoptotic cells can cause inflammation. And dysfunction of skin caused by inflammation results in inability of pathogens clearance. Thus, skin infectious diseases, inflammatory diseases and autoimmune diseases interact tightly with each other. The solid lines represent known functions, while dashed lines represent speculations.

Recent studies have shown that several collectins, including MBL, SP-A, SP-D, CL-L1, and CL-K1, can be detected in skin tissue (38, 39). Multiple functions of these collectins in skin immune homeostasis have been discovered (**Figure 4**). And the relationships between collectins and skin diseases have also been reported (40). Hence, these five collectins are discussed below.

### Mannose-Binding Lectin

**Figure 5:**
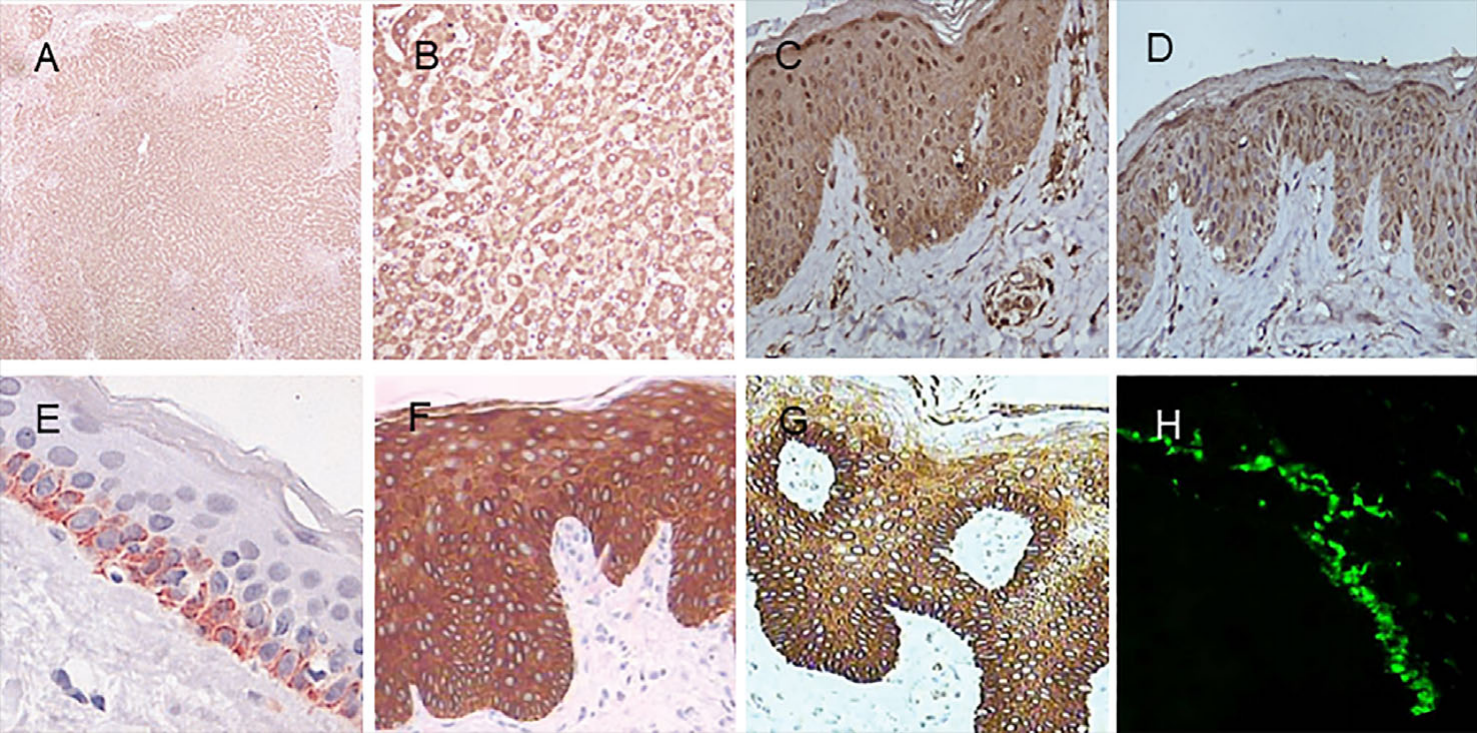
Immunohistochemistry of MBL, SP-A, and SP-D in normal human tissues. **(A)** MBL in the liver (DAB staining with MBL2 polyclonal antibody, 40×). **(B)** MBL in the liver (MBL2 polyclonal antibody, 200×). **(C)** MBL in the skin (MBL2 polyclonal antibody, 400×). **(D)** SP-A in the skin (SFTPA1 polyclonal antibody, 400×). **(E)** SP-D in the skin (Hyb 245-1,400×). **(F)** SP-D in psoriasis lesions (monoclonal antibody Hyb 245-1, 400×). **(G)** SP-D in atopic dermatitis (monoclonal antibody Hyb 245-1, 400×). **(H)** Deposition of MBL in the basal membrane zone with granular pattern by immunofluorescence. Panel **(E)** was modified and reprinted from Madsen, et al. in 2015 (43). Panel **(F, G)** are modified and reprinted from Hohwy, et al. in 2006 (44). Panel **(H)** was modified and reprinted from Wallim LR et al. in 2014 (45).

MBL is encoded by a gene on chromosome 10q11.2-21 called *MBL2*. Humans lack the MBL-A form, derived from the MBL1 gene present in other species. Polymorphisms in coding and promoter regions of *MBL2* result in variations that affect MBL circulation levels (41). MBL is synthesized mainly by the *liver* and released into the serum, but is also found in skin lesions of pemphigus (42). Immunohistochemistry shows that MBL is diffusely positive in the normal human *liver* tissue. Staining intensity is pronounced in the cytoplasm of hepatocytes. (**Figure 5**).

MBL has a variety of functions. It binds to pathogens and forms complexes with MBL-associated serine proteases (MASPs) that initiate the lectin pathway of the complement system, resulting in enhanced microbial clearance *via* opsonization (46). MBL binds to apoptotic cells in the skin, including Jurkat cells and human monocyte-derived macrophages, leading to apoptotic cell clearance (47). MBL also shows two-way biological effects in terms of modulating inflammatory reactions by inducing cytokine secretion. On the one hand, in the absence of microbes, MBL enhances the production of pro-inflammatory cytokines IL-6, IL-8, and monocyte chemoattractant protein-1 in human umbilical vein endothelial cells, as well as TNF-α in a dose-dependent manner in human corneal epithelial cells, when challenged with microorganisms (48, 49). On the other hand, MBL inhibits the secretion of pro-inflammatory mediators IL-1α and IL-1β and increases the production of anti-inflammatory mediators IL-10 and IL-1α in human monocytes, which illustrates its role in alleviating inflammatory reactions during apoptotic cell clearance (50). Thus, MBL usually regulates inflammatory cytokines positively during anti-microbial responses but negatively during apoptosis to avoid autoimmunity.

Abnormal MBL levels occur when immune deficiencies, infections, or allergic reactions involve the skin. MBL deficient patients are more susceptible to recurrent cutaneous abscesses, recurrent folliculitis, and sporotrichosis (40, 51, 52). MBL participates in the defense against bacterial, viral, and fungal infections. However, the role of MBL deficiency in recurrent skin infections including cutaneous abscesses and folliculitis has not been entirely investigated. A few studies have used murine models to analyze the function of MBL in the skin. Møller-Kristensen and colleagues observed that MBL knockout mice showed epidermal acanthosis on histologic examination and that MBL-null mice exhibited delayed spontaneous separation of eschars (47). A later study comparing wild-type and MBL knockout mice in a burn model showed that murine MBL deficiency increased the host’s susceptibility to *Pseudomonas aeruginosa* infection; the skin bacteria load in the MBL-null mice was not different from that in the wild-type mice, but IL-6 and TNF-α levels were lower in the MBL-null mice (53). It seems that the skin has other methods of inhibiting local infections when MBL is lacking, but MBL has a positive influence on cytokine production during pathogen invasion. Thus, lack of MBL and MBL-mediated clearance of apoptotic cells lead likely to pro-inflammatory responses. *MBL2* polymorphism has been associated with tuberculosis susceptibility (54). MASP-2, the downstream protease activated by MBL, has been shown to exhibit a protective effect by upregulating IL-2 and IFN-γ in a rabbit model of cutaneous tuberculosis (55).

MBL also plays a key role in autoimmune skin diseases, such as SLE, dermatomyositis, and pemphigus vulgaris (42, 56, 57). SLE mechanisms involve apoptosis, complement activation, and infection. The association between MBL and SLE is complex, as some studies results are contradictory. Indirectly, MBL was shown to bind to apoptotic keratinocytes after ultraviolet B irradiation in an *in vitro* experiment, suggesting that a lack of MBL leads to a buildup of apoptotic debris in the skin, thus allowing the development of autoimmune diseases like SLE (47). In contrast, MBL dysfunction lowers a host’s resistance to infection, which might trigger SLE. MBL deficiency might therefore increase the risk of SLE by incorrect clearance of apoptotic cells, and by increasing skin infection rate leading to frequent inflammation as well. Further, MBL and other components of the complement system were found by immunofluorescence to be deposited around skin lesions, rather than in normal tissues, in SLE patients (45). Locally increased levels of MBL and other components are suggestive of their roles in complement activation, which might be a cause of cell destruction. Some of the reasons for these contradictory results might be attributed to different study backgrounds or the complex nature of SLE. Like tissue deposition, serum MBL levels in SLE patients appear to be inconsistent in different studies. Panda et al. found that *MBL2* polymorphism leading to low MBL production in the serum causes susceptibility to SLE (58), whereas a previous study comparing 93 SLE and 67 healthy controls showed that the plasma MBL levels in SLE patients were higher than in healthy people (53). Some studies have shown that MBL levels increase moderately (between 1.5- and three-fold) during acute phase responses (59), but the specific role of MBL in SLE occurrence and development has not yet been identified. Like SLE patients, pemphigus patients show MBL deposits in the basal membrane zone of the epidermis in a study, which seems to cause conflicts between apoptosis and complement activation (42). In this study, C3 and C5b-9 were found colocalized with MBL, indicating that MBL has a role in the pathogenesis of pemphigus. MBL deficiency is linked with dermatomyositis, which supports the concept that apoptosis dysfunction results in autoimmunity (57).

MBL participates in chronic cutaneous inflammatory diseases, including psoriasis, lichen planus, Behçet’s disease, cellulitis, and ulcerations (40). MBL promotes keratinocyte production of chemokine (C-X-C motif) ligand 1 and enhances neutrophil infiltration in psoriatic lesions (6). Genotypes associated with low MBL production lead to high susceptibility to oral lichen planus (60). *MBL2* polymorphism has been linked with Behçet’s disease, cellulitis, and ulcerations (40). Moreover, MBL may be associated with the modulation of the production of cytokines (including IL-6, lipopolysaccharide-induced CXC chemokine, monocyte chemoattractant protein-1, and macrophage inflammatory protein-2), cell adhesion molecules (including L-selectin and P-selectin), and insulin-like growth factor-binding protein-3, indicating its role in cutaneous inflammatory diseases (47).

### SP-A and SP-D

SP-A has 2 functional genes in humans, *SFTPA1* and *SFTPA2*, encoding SP-A1 and SP-A2, respectively. SP-D is encoded by *SFTPD*. These genes are all located on chromosome 10, together with *MBL2*. Both SP-A and SP-D are synthesized by alveolar epithelial type II cells and non-ciliated respiratory epithelial cells (Clara cells) (61). SP-D seems to appear more widely distributed than SP-A. They both have been detected in the female reproductive tract as well as in the urinary tract, gastrointestinal tract, eye, nose, ear, central nervous system, coronary arteries, and epidermis. Results of PCR and WB also showed that they are synthesized locally (62).

Some immunohistochemistry studies have reported different locations of SP-A and SP-D in human skin. In a rather early study by Mo et al., SP-A appeared in the stratum corneum and stratum granulosum, while SP-D was distributed in all epidermal layers and the dermis (37). Aiad et al. found that SP-A was mainly detected in the basal layer, which was agreed upon by Akman (63, 64). SP-D was found to be restricted in the basal layer, rather than diffusely spread, in later research by Hohwy et al. (44). Madsen and colleagues showed that SP-A and SP-D were positive in the basal layer of skin (43). The use of different antibodies or research methods might explain these differences. The consequences conducted by monoclonal antibody by Madsen and colleagues seem to be more specific than those conducted by polyclone antibodies.

As an innate immunity organ, the skin is the first line of defense. SP-A and SP-D recognize microbial carbohydrate ligands and form massive aggregates that prevent microbial colonization and help remove pathogens (61). Regarding the structure of their carbohydrate recognition domains, SP-A and SP-D recognize pathogens as pattern recognition molecules and modulate cell immune functions. SP-A binds to calreticulin, CD14, and toll-like receptor 2, among other receptors, and mediates the phagocytosis of microorganisms (65). SP-D binds to CD14 and signal regulatory protein alpha, among other receptors, and mediates the inhibition of cytokine release (2). SP-A and SP-D bind to and may agglutinate a broad range of pathogenic microorganisms, including bacteria, viruses, fungi, and yeasts. Both proteins increase neutrophil uptake of *Escherichia coli, Streptococcus pneumoniae*, and *Staphylococcus aureus* and can increase the permeability of the microbial cell membrane to inhibit the growth of gram-negative bacteria (66). In absence of microbial ligands, SP-A and SP-D bind *via* their collagen tail to SIRP-α and mediate anti-inflammatory response. In presence of microbial ligands, they bind *via* other receptors (opsonizing) and contribute to a pro-inflammatory response.

SP-A and SP-D participate also in skin inflammatory diseases, such as psoriasis. SP-A and SP-D have both been found to be diffusely positive in the epidermis of psoriatic lesions by immunohistochemistry (44, 63). This phenomenon is not involved with stronger staining intensity but more with increased numbers of stained cells. The pathogenesis of psoriasis has been attributed to the activation of lymphocytes and cytokines. SP-A and SP-D modulate the functions of antigen-presenting cells, including macrophages and dendritic cells, which may lead to IL-12-dependent Th 1 responses (67, 68). TNF-α contributes a lot to the pathogenesis of psoriasis. The variation trend of TNF-α in serum has been found corresponding with those of SP-A and SP-D (44, 63). As in psoriasis, immunohistochemistry shows high levels of SP-A and SP-D in the skin lesions of atopic dermatitis, lichen planus, and Behçet’s disease (64). Despite increased levels of surfactant proteins in psoriasis, their functions in inflammation are complex. SP-A and SP-D inhibit inflammatory responses in non-inflammatory conditions but further activate nuclear factor kappa B signaling and pro-inflammatory mediator production in inflammatory conditions (69). SP-A and SP-D also suppress inflammation *via* SIRP-α on macrophages and epithelia, while stimulating inflammation by binding calreticulin/CD91 (70). Thus, the specific roles of SP-A and SP-D in psoriasis needs further investigation.

SP-A and SP-D may participate in autoimmune skin diseases. Serum SP-D levels in SLE patients have been shown to be lower than in healthy people (7). This may be because both collectins can recognize DNA from apoptotic cells and mediate phagocytosis (71). However, SP-A and SP-D suppress apoptotic cell clearance by binding SIRP-α on macrophages (72). Besides, both surfactants prevent cells from apoptosis by activating the phosphatidylinositol 3-kinase/protein-serine-threonine kinases signaling pathway and the dephosphorylation of forkhead transcription factors (72), which might also be the roles of these collectins in autoimmune skin diseases.

### CL-L1 (Collectin-10)

CL-L1 and CL-K1 were discovered when screening expressed sequence tag databases for homologs of MBL (73, 74). The encoding gene (*COLEC10*) is located on chromosome 8q23-q24.1 in humans. About 20 variants have been identified in the promoter regions, exons, and flanking regions of *COLEC*10 (75).

**Figure 6:**
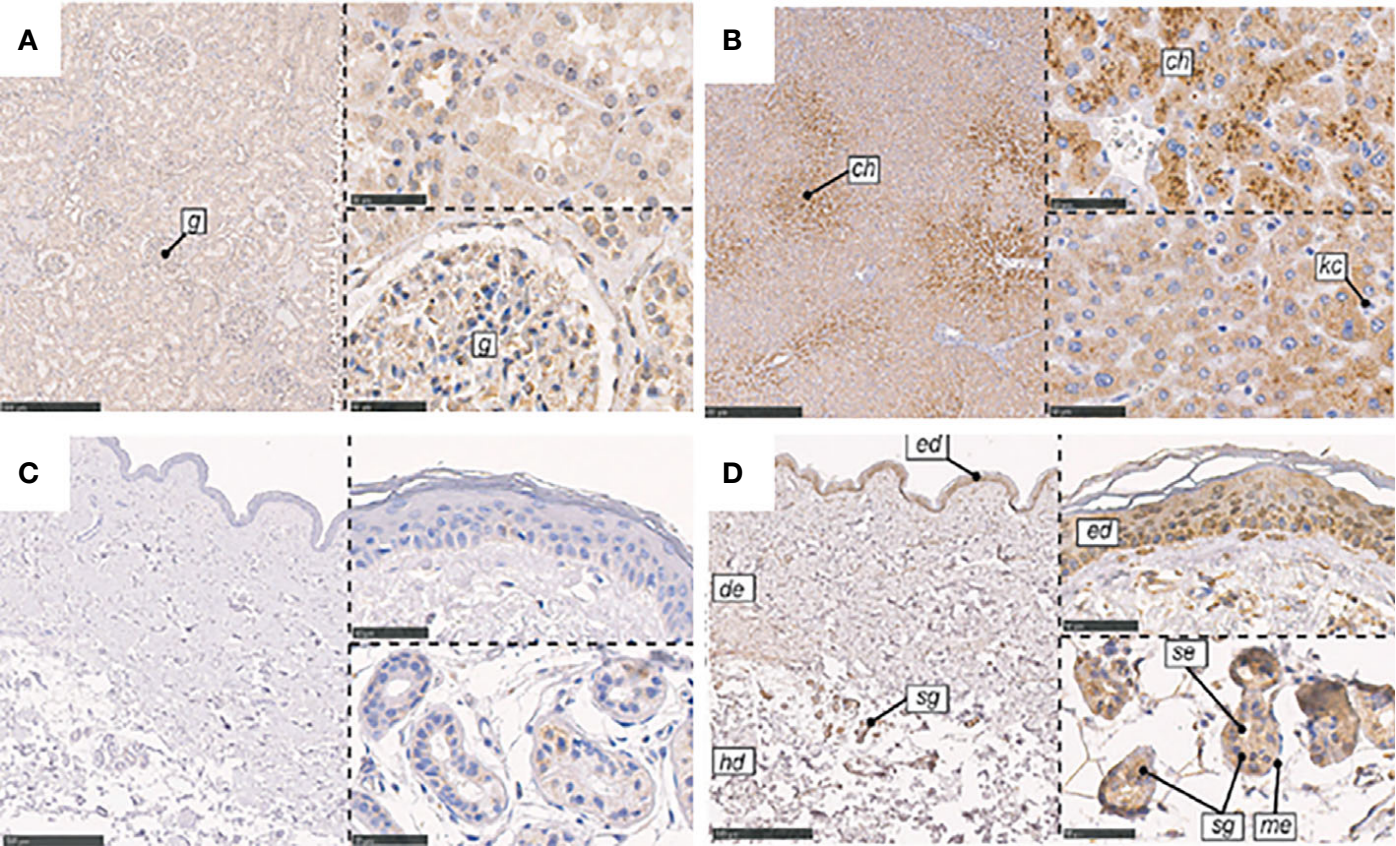
Immunohistochemical localization of CL-K1 and CL-L1 in formalin-fixed and paraffin-embedded sections of kidney, liver, and skin. **(A)** CL-K1 in the kidney. **(B)** CL-L1 in the liver. **(C)** CL-K1 in the skin. **(D)** CL-L1 in the skin. Italic letters within the images refer to: 1) h: hepatocytes, ch: centrilobular hepatocytes, and kc: Kupffer cells for the liver; 2) g: glomerulus for the kidney; and 3) ed: epidermis, de: dermis, hd: hypodermis, sg: sweat glands, me: myoepithelial cells, and se: stratified cuboidal epithelia cells for the skin. This figure is modified and reprinted from Hansen, et al. in 2018 (36). Scale bars in large sections correspond to 500 μm and in small sections to 50 μm.

CL-L1 was found to be ubiquitously expressed in human tissues, including liver, heart, spleen, stomach, prostate, and adrenal (74). CL-L1 mRNA has also been detected in the liver, stomach, and embryos of mice (76). Although the epidermis contains low CL-L1 mRNA levels, IHC study demonstrated its location in the skin (38). While CL-L1 is strongly positive in centrilobular hepatocytes in the liver, it is weakly positive in the epidermis, though it is also strongly positive in sweat glands and ducts (**Figure 6**).

It is likely that the initial reports on the ligand specificity of CL-L1, demonstrating preference for mannose-like carbohydrate, were influenced by its complex formation with CL-K1 (77, 78). Unlike other collectins, CL-L1 has four C-terminally located lysine residues in the CRD, which might function as signals for translocation from cytoplasm to nucleus (79). Though the role of CL-L1 in complement activation has not been proven, the heteromer formed by CL-L1 and CL-K1 (named CL-LK) has been noticed for its function of activation of the lectin pathway *via* MASP-2 in serum (80). Despite that homomeric CL-L1 forms complexes with MASP-1 products, its role in activation of the lectin pathway has not been proven.

In an investigation of the roles of lectin pathway components in 332 patients with recurrent infections, serum CL-L1 levels quantified by time-resolved immunofluorometric assay in 25 patients with abscesses were 15% higher than in healthy people (81). However, low serum CL-L1 levels are associated with the increased the risk of nosocomial infections in children (82). Despite the lack of evidence of CL-L1 binding to microorganisms independently, these results suggest that CL-L1 not only helps in the defense against pathogens, but also plays a role in inflammation (83). Serum CL-L1 levels are lower in SLE patients than in healthy individuals. Meanwhile, CL-L1 in serum has a significantly negative correlation with SLE activity (83). The possible explanation for this might be that CL-L1 is consumed in inflammatory processes or binds to apoptotic cells. CL-L1 may play a similar role in inflammatory and autoimmune skin diseases.

### CL-K1 (Collectin-11)

The gene encoding CL-K1, *COLEC11*, comprises seven exons and is located on chromosome 2p25 (73). CL-K1 is present in most tissues. Through reverse transcription polymerase chain reaction, CL-K1 mRNA has mainly been detected in the *kidney, liver*, and *heart* of humans, with some but less detected in the *bone marrow and muscle* (38, 83). Immunohistochemistry shows pronounced CL-K1 in the tubular system of the kidney in humans (**Figure 5**). Although CL-K1 content in the skin is not as great as in the kidney, this secreted protein is positive in the stratum basale and sweat glands but weakly positive in myoepithelial cells (38).

CL-K1 circulates in the blood in form of heteromeric complexes with CL-L1 (CL-LK). Deficiency in either CL-L1, CL-K1 or MASP-3 lead to 3MC syndrome (a syndrome characterized by hypertelorism, blepharophimosis, ptosis, cleft lip, cleft palate, and other ectoderm manifestations) (84–86). Plasma CL-K1 levels are below in 3MC syndrome patients, while they are around 300 ng/ml in healthy individuals (84). The possible role of CL-K1, CL-L1 and MASP-3 in the 3 MC syndrome is hypothesized to be associated with neural crest cells migration (87). Melanocytes originate also from neural crest cells and contribute to skin immunity. As the skin is an ectoderm organ, CL-L1 or CL-K1 dysfunction might also be associated with defects in skin immune homeostasis.

CL-K1 binds to L-fucose and D-mannose and interacts with nucleic acids (77, 88, 89). This helps with the function of CL-K1 in the lectin pathway as a pattern recognition receptor to bind to bacteria, such as intact *E. coli, Candida albicans*, and *P. aeruginosa*, and yeast extracts (73). A study of CL-K1 knockout mice showed that a lack of CL-K1 leads to susceptibility to *S. pneumoniae* infection (90). *S. pneumoniae* has been isolated as a major pathogen carried by the skin (91). Therefore, CL-K1 possibly participates in the defense against pathogens in the skin. It may also affect skin immunity through these such pathogens.

Serum CL-K1 levels are higher in SLE patients with discoid skin manifestations than in those without discoid lesions (83). Similar to CL-L1, serum CL-K1 shows a significantly negative correlation with SLE activity. The positive rate of anti-CL-K1 antibody is very high in patients with SLE in whom both anti-dsDNA and anti-Sm antibodies are negative, illustrating its value in the diagnosis of SLE (92). Though the specific mechanisms of CL-K1 in SLE are not clear, CL-K1 may function in the opsonophagocytosis of apoptotic cells by binding to calreticulin (a coreceptor for CD91) like other collectins, such as MBL, SP-A, and SP-D (42).

CL-K1 can modulate cytokine production. It increases IL-10 production but decreases IL-6 production in retinal pigment epithelial cells *via* signal regulatory protein α (93). In addition, dermal fibroblasts have been reprogrammed into induced pluripotent stem cell-derived retinal pigment epithelium cells (94). Based on the similarities between these two cell types, CL-K1 may be considered to act as a modulator of skin inflammation. IL-10 and IL-6 are also involved in keloids, wound healing, and some skin diseases (95, 96). Thus, CL-K1 might take part in skin inflammatory responses by affecting fibroblast activity.

## Therapeutic Strategies Targeting Collectins and Relevant Regulators

Since collectins participate in the pathogenesis of various diseases, the promotion of their secretion or inhibition can be used as therapeutic strategies. Besides, collectin substitution is also an available therapeutic method. As for inhibition, there are usually three possible ways to suppress the function of pattern recognition molecules in the complement system: 1) prevent the binding of molecules to target molecules; 2) prevent the binding of associated serine proteases to molecules; and 3) prevent the conformational changes of molecules that are necessary for the activation of serine proteases (97). The feasibility of using the third method remains to be studied, as MASPs is found in complex with collectins prior to the binding to activating carbohydrate ligands.

MBL substitution treatments have been studied for years. There are two main MBL substitutive proteins: plasms-derived MBL (pdMBL) and recombinant MBL (rMBL). Both have been tested on patients with MBL deficiency in clinical trials, and the results seem to be positive (98). However, rMBL appears to have advantages over pdMBL, as pdMBL restores lectin pathway function suboptimally, despite restored plasma MBL levels (99). This may be because rMBL can associate with free non-bound MASPs, while pdMBL has pre-activated MASPs, which are inactivated immediately (100). Though MBL replacement therapy is still under debate, it brings good news to patients with MBL deficiency. It might be a choice for the treatment of skin diseases associated with *MBL2* polymorphism, such as cutaneous abscesses, recurrent folliculitis, and other inflammatory skin diseases.

Monoclonal antibodies that block the globular domains of collectins can efficiently interfere with ligand binding. Anti-MBL antibodies have been successfully used to inhibit lectin pathway activation and inflammation (101). The 3F8 anti-MBL-2 monoclonal antibody was administered to a murine model of atypical hemolytic uremic syndrome and was shown to attenuate C3d deposition and renal injury, suggesting that *MBL2* inhibition is a mitigating factor *in vivo* (102). Mab 3F8 has also been shown to protect against heart infarction and occlusive arterial thrombogenesis in a murine model of myocardial ischemia (103). Polyman 9, which is a selective mouse MBL-C inhibitor, can effectively reduce circulating C4b levels and sensorimotor deficits and promote neurogenesis and astrogliosis in experimental traumatic brain injury (104). These inhibitory agents targeting MBL may inspire treatments for autoimmune skin diseases related to complement activation, such as pemphigus vulgaris.

MASPs participate in the lectin pathway and form complexes with collectins. They are relevant to cutaneous tuberculosis, hereditary angioedema, and scabies. MASP-1/2 inhibition can block the lectin pathway from an early stage. An anti-MASP-2 antibody (OMS721) is in clinical trials for the treatment of atypical hemolytic uremic syndrome and other thrombotic microangiopathies, as well as IgA nephropathy, lupus nephritis, membranous nephropathy, and C3 glomerulopathy (95). MBL/ficolin-associated protein-1 (MAP-1, alias Map44) is a potential endogenous inhibitor of the lectin pathway that functions by displacing MASP-2 and inhibiting MBL-dependent complement activation. It can attenuate myocardial injury and thrombogenesis in murine myocardial ischemia and reperfusion models (105). Two *Schistocerca gregaria* protease inhibitors have been proven to block the lectin pathway *in vitro* (106). Thus, these MASP inhibitors may also effectively block abnormal complement pathway activation and attenuate tissue injury in skin diseases.

Collectins modulate the downstream molecules of the lectin pathway. C5 is an important member of the complement system, which is the cleavage result from the lectin pathway. C5 accumulation causes tissue injury in autoimmune diseases, such as pemphigus. Pexelizumab is a recombinant humanized single-chain antibody fragment that prevents the cleavage of C5, which is the downstream of the lectin pathway (107). Though pexelizumab in patients with segmentation T elevation myocardial infarction has attenuated the increase of IL-6, it has no impact on other inflammatory molecules. The effect of C5 monoclonal antibody need to be further investigated in skin diseases.

Collectins also participate in inflammatory reactions by interacting with TNF-α (63), though the precise mechanism remains unknown. Infliximab, which is a TNF-α monoclonal antibody applied in psoriasis treatments, appears to inhibit SP-D in murine models of intestinal ischemia/reperfusion or lung injury and relieve tissue injury by decreasing SP-D levels (108). Based on these findings, infliximab might be effective in skin diseases like atopic dermatitis in which SP-D and other collectins participate. The biological agents targeting collectins and relevant regulators are summarized in **Table 1**.

**Table 1:** Biological agents targeting collectins and relevant regulators.

Biological agent	Type	Effect	Model	Result	Reference
pdMBL	Plasma-derived protein	Substitutes MBL	Patients	Restores plasma MBL levels, partially restores LP function	(98)
rMBL	Recombinant protein	Substitutes MBL	Patients	Restores plasma MBL levels, perfectly restores LP function	(98)
3F8	Monoclonal antibody	Inhibits MBL	Murine model of STEC-HUS	Attenuates C3d deposition and renal injury	(102)
3F8	Monoclonal antibody	Inhibits MBL	Murine model of myocardial ischemia/reperfusion	Preserves myocardial injury, reduces infarct size, prevents arterial thrombogenesis	(103)
Polyman 9	Polymannosylated compound	Inhibits MBL	Murine model of controlled cortical impact traumatic brain injury	Improves sensorimotor deficits, promotes neurogenesis and astrogliosis	(104)
SFMI-1	Peptide	Inhibits MASP-1	In vitro	Prevents C3 and C4 deposition	(106)
OMS721	Monoclonal antibody	Inhibits MASP-2	Patients with IgA nephropathy, lupus nephritis, membranous nephropathy, or C3 glomerulopathy	In progress	(97)
MAP-1	Endogenous inhibitor	Inhibits MASP-2	Murine model of myocardial ischemia/reperfusion	Attenuates myocardial injury and thrombogenesis	(104)
SFMI-2	Peptide	Inhibits MASP-2	In vitro	Prevents C3 and C4 deposition	(106)
Pexelizumab	Monoclonal antibody	Inhibits C5	Patients with ST-segment elevation myocardial infarction	Blunts the elevation in C5a, attenuates the increases of IL-6	(107)
Infliximab	Monoclonal antibody	Inhibits TNF-α	Murine model of intestinal ischemia/reperfusion	Protective effect on damage, decreases serum SP-D levels	(108)
Infliximab	Monoclonal antibody	Decreases SP-D	Acute lung injury	Preventive effect on damage, decreases serum SP-D levels	(108)

LP, lectin pathway; STEC-HUS, Shiga toxin-producing Escherichia coli hemolytic and uremic syndrome.

## Conclusions and Prospective Views

Collectins participate in the initiation of the lectin pathway of the complement system, which is necessary for the defensive functions of the skin. Additionally, collectins contribute to opsonization and inflammatory reactions in skin diseases. Although the functions of collectins still need to be further investigated, many studies have shown that collectins help to maintain skin immune homeostasis and prevent skin diseases. Therapeutic strategies targeting collectins or relevant regulators are limited in their clinical applications because they are still being developed and undergoing trials. However, replacement therapies and complement inhibition for certain diseases have so far shown positive, often satisfactory results. Further studies should be performed to reveal the precise mechanisms underlying collectin regulation of skin immune homeostasis and to explore potential approaches to rectify collectin abnormalities in skin diseases.

## Author Contributions

TW wrote the first draft of the manuscript. KL and SX contributed to the conception of the review. YX conceived the central idea and finalized this paper. All authors contributed to the article and approved the submitted version.

## Funding

This study was supported by the Innovation Capability Support Plan of Shaanxi Province (No.2019TD-034).

## Conflict of Interest

The authors declare that the research was conducted in the absence of any commercial or financial relationships that could be construed as a potential conflict of interest.

## Abbreviations

CL-K1, collectin kidney 1; CL-L1, collectin liver 1; CL-P1, collectin placenta 1; IL, interleukin; MASP, mannose-binding lectin associated serine protease; MBL, mannose-binding lectin; SLE, systemic lupus erythematosus; SP-A, surfactant protein A; SP-D, surfactant protein D; TNF-α, tumor necrosis factor-α.
